# Small-scale and backyard livestock owners needs assessment in the western United States

**DOI:** 10.1371/journal.pone.0212372

**Published:** 2019-02-14

**Authors:** Alda F. A. Pires, Amos Peterson, Jerome N. Baron, Ragan Adams, Beatriz Martínez-López, Dale Moore

**Affiliations:** 1 Department of Population Health and Reproduction, School of Veterinary Medicine, University of California-Davis, Davis, California, United States of America; 2 Department of Veterinary Clinical Sciences, College of Veterinary Medicine, Washington State University, Pullman, Washington, United States of America; 3 Center for Animal Disease Modeling and Surveillance, Department of Medicine and Epidemiology, School of Veterinary Medicine, University of California-Davis, Davis, California, United States of America; 4 Veterinary Extension Specialist, Department of Clinical Sciences, College of Veterinary Medicine and Biomedical Sciences, Colorado State University, Fort Collins, Colorado, United States of America; Swedish National Veterinary Institute, SWEDEN

## Abstract

The number of small-scale and backyard livestock and poultry owners in urban and peri-urban areas has increased greatly over the last 10 years in the U.S. However, these animal owners may live in areas without access to livestock and/or poultry veterinary care. The purpose of this study was to identify potential veterinary service needs of these animal owners in the western US, assess their use of management and husbandry practices with regards to disease prevention, and assess their attitudes about animal health and food safety. A semi-structured survey was made available to small-scale and backyard livestock and poultry owners in Washington State, California, Colorado and Oregon. The survey instrument included questions about types of animals reared, uses of the animals, veterinary services and information-seeking behaviors of owners, attitudes on animal health and food safety, and management practices. Four hundred thirty-five individuals completed at least some portion of the survey. Most described themselves as living in rural areas (76%). Most (86%) owned chickens, 53% owned small ruminants, and 31% owned cattle. Many individuals owned more than one species and most had fewer than 20 animals of a given species. About 74% of respondents utilized their animals’ products for their own consumption but 48% sold animal products (primarily through internet sales (35%) or farmers’ markets (25%)). Overwhelmingly, respondents gained information about animal health (82%) and animal treatment procedures (71%) from the internet. Respondents reported their veterinarian’s practice type as companion animal (26%) or a mixed animal or food animal predominant (66%). Overall, respondents were very satisfied with the level of care (82%), but 43% had not sought animal health care in last 12 months. However, the veterinarian’s primary practice type and owner’s satisfaction with veterinary care were associated with their location (state), species owned, and urban or peri-urban setting. Livestock species type (cattle, small ruminants and swine), and use (personal or commercial) were associated with implementation of different biosecurity practices. The results of this survey highlight some of the needs of these animal owners for veterinary care and information which are location- and species-specific. Veterinary care for these small-scale and backyard animals is vital to the health and welfare of the animals as well as for identification of zoonoses and assurance of the food safety of animal products.

## Introduction

The number of owners of peridomestic or backyard livestock and poultry is increasing in urban and peri-urban areas, potentially increasing risks for zoonoses and posing challenges for veterinary practices in these areas [1–7]. Historical data from the United States Department of Agriculture (USDA) indicates that from 1988 to 2007, the level of urban and peri-urban agriculture (UPA), both plant and animal, increased from 30% to 40% [8].

The potential risk of public health and zoonotic disease risk from greater contact at the human-animal interface includes transmission of zoonoses such as *Salmonella* and Highly Pathogenic Avian Influenza (HPAI). HPAI was identified in 21 backyard poultry flocks in 11 states in 2015, illustrating the potential for future HPAI transmission and outbreaks in this population [9]. Without adequate preventive medicine services and access to veterinary care, these owners and their animals may be at higher risk than commercial poultry flocks [2]. Public perception of disease risk, particularly zoonoses, varies with husbandry practices and experience. Some researchers assert that small-scale and backyard poultry flocks are not at greater risk, while others have shown that backyard flocks do increase exposure to diseases such as salmonellosis [2,10]. Another potential problem is that backyard animals may be reservoirs of disease for commercial stock. Some virulent Newcastle Disease (vND, formerly known as Exotic Newcastle Disease) and Marek’s Disease outbreaks have originated in backyard flocks and spread to commercial flocks [3,11]. To identify these pathogens early, owners should have built a valid Veterinarian-Client-Patient Relationship (VCPR) with their local veterinarians [12,13].

Small-scale and backyard poultry and livestock owners have been previously surveyed to identify knowledge gaps, animal husbandry awareness, and for census reasons [2,5,11,14–21]. These studies reported that although this population is difficult to assess, some common concerns and issues associated with UPA livestock/poultry ownership could be identified. First, although poultry presence in four major cities (Los Angeles, Denver, Miami and New York City) was low, approximately 4% of people anticipated owning poultry within the next 4 years [22]. Second, the belief that backyard-raised chickens and eggs were healthier when compared to commercial egg products was widely held. A 2014 study of poultry owners conducted in collaboration between Master Gardener programs and *Better Homes and Gardens* magazine found that a significant proportion of respondents identified themselves as urban or suburban, 41% had incomes greater than $100,000, and the majority of these owners raised poultry for their own use [14]. These results are consistent with other studies showing that the majority of backyard chicken owners utilize their poultry products for private use [2,17,23]. Moreover, many owners view poultry more as pets than production animals [2,14]. As no national standards for care or licensure for backyard poultry or livestock owners exist, the potential welfare of and disease risks for these animals should be evaluated [24]. As people become more attached to their animals, their motivation to seek out veterinary care may also increase. Many survey respondents indicated a desire for more and better-trained providers to care for their poultry [14]. Considering that the majority of urban and peri-urban veterinary practitioners are likely focused on small animal (i.e., companion animal) medicine, exactly where these poultry and livestock owners can find proficient livestock care is an open question.

A 2006 article on the “Future of Veterinary Medicine in Poultry Production” described the current educational environment as one in which a small consolidated group of veterinary colleges have maintained robust poultry medicine programs and the rest have either eliminated or lack any poultry medicine programs whatsoever [25]. This has not resulted in a serious veterinary shortage for the commercial poultry industry because it has developed private and independent post-graduate in-house training programs. However, in light of the dearth of collegiate programs, providing clinical training in poultry medicine would require a revamp of current veterinary curriculae in most colleges [25]. In addition, new US FDA requirements for veterinary oversight of in-feed and water antibiotics and the need for a valid VCPR should increase the demand for veterinary services by small-scale livestock and poultry owners [12]. On January 1, 2017, the US FDA’s GFI213 was fully implemented: the in-feed use of medically important antimicrobials for growth promotion was removed from product labels, and a medically important antimicrobials now required veterinary oversight for use (a veterinary feed directive for antibiotics administered in feed and veterinary prescription for antibiotics used in water) [12]. The question arose whether or not small-scale animal/ UPA producers experience a lack of access to adequate veterinary care. Therefore, we conducted a cross-sectional study to assess the knowledge of small-scale and backyard livestock owners in four states (Washington, California, Oregon and Colorado) regarding animal health, biosecurity, food safety, husbandry and hygiene practices and their perceptions of veterinary care needs for their animals.

## Materials and methods

### Study population and survey instrument

The semi-structured survey consisted of 28 questions, including a combination of binary, categorical and open-ended questions, and was delivered using an online survey platform and in person (the survey is available from the corresponding author). A pilot survey was distributed at the Country Living Expo in Stanwood, WA, and was adapted for this study. The final survey tool was pre-tested with five members of the faculty and staff at University of California, School of Veterinary Medicine–Davis. The survey instrument was approved for use with human subjects by the Institutional Review Board, University of California, Davis (#726301–1), and Washington State University, Pullman (#14537). The survey was introduced at The National Heirloom Exposition, September 8–10, 2015, Santa Rosa, CA, and at the UCD Goat Day, UC Davis, CA, January 23, 2016. Hard copies of the survey were made available at these events and by request to any interested owners during the study period. If individuals expressed interest in the survey but did not have time to complete it, they were given a card with a link to an online survey. The online survey was advertised in the four states as follows: flyers that were posted at local feed stores, fairs and conferences; social media (e.g., Facebook, Twitter); newsletters; email lists; local and regional small-scale holder interest group Web sites; and word-of-mouth. The cover letter described the selection criteria for production animal species (poultry, cattle, swine, sheep, goats, or camelids), small-scale farm/premise size (operations with annual sales of less than $500,000; maximum of 500 goats/sheep, 100 cows or 100 pigs; or are poultry producers who process or sell less than 1,000 chickens a year) and peri-urban/backyard premises (animal-raising within urban and peri-urban, residential areas with the goal of production of animal products for self-consumption and/or distribution and marketing of products within those areas). The survey was available to respondents between September 8, 2015 and June 30, 2016 in California, Oregon, and Washington, and between November 15, 2016 and January 15, 2017 in Colorado.

The survey tool focused on animal health (including veterinary care and health needs), husbandry, biosecurity, animal products and food safety, and demographics. Comments were invited at the conclusion of the survey. The first section asked respondents to identify a veterinarian in their area willing to treat poultry and/or livestock (commercial and non-commercial animals) and asked if or when they would seek care for their animals. Respondents were queried on their knowledge of local veterinarians, their level of satisfaction with current services and what services they would like in the future. They were asked if their animals had experienced a significant health concern in the previous 12 months and, if so, had they sought veterinary care. To determine why owners might choose not to call their local veterinarian, a follow-up question concerning barriers to seeking care was included. Respondents were asked about biosecurity practices including wildlife contact, use of personal protective equipment, tools and equipment sharing, acquisition of new animals and quarantine.

Participants were asked about what resources they typically utilized to obtain both medical and animal husbandry information and the frequency with which they did so. They were also queried on numbers of animals they owned and whether their neighbors owned livestock and/or poultry. The participants were asked about their use of animals and animal products, food safety and hygiene when handling animal products, and level of comfort performing a series of typical animal health procedures.

### Data management and analysis

All data were collected anonymously. Data were downloaded or entered into Microsoft Excel spreadsheets (Microsoft, Redmond, WA). To maintain confidentiality, no information was retained that linked individual respondents to their survey data. Response categories were typically binary, multinomial, or categorical. For open-ended queries and qualitative responses, all of the responses were read for themes that were added to the database for those questions.

Descriptive analysis (i.e., frequencies and percentages) and graphic representation, were done using Stata/IC version 14.2 (StataCorp, College Station, Texas, USA) and Excel (Microsoft Corp., Redmond, WA). Mapping of premises was done by using a United States Counties polygon map. Each county was given a numerical attribute representing the number of premises it contained in ArcGIS, to map premise distribution (Environmental Systems Research Institute (ESRI), ArGIS Release 10.5, Redlands, CA).

Most questions in the survey were categorical and analyzed using frequency tables and graphs, as well as logistic regression. A number of questions relating to why an owner might seek help for an animal health problem, and what problems they had encountered in the past, were open-ended. These answers were categorized in broader groups by frequency. Health problems were categorized by disease type, anatomical system and animal type. Consideration for seeking veterinary help was broadly categorized in terms of cost, owner’s assessment of a problem, owner’s knowledge and availability of experienced veterinary help. Responses from 13 individuals from neighboring states to the four states of interest, and 5 not stating which states they came from where excluded from further statistical analysis.

Logistic regression was conducted in R (version 3.3.1, R Core Team). For the logistic regression, we considered 8 outcome variables (presence of veterinarian who treats treating livestock, owner’s veterinarian primary practice type, satisfaction with veterinary care received, quarantine practices for new animals and sick animals, allowing contact of livestock with wildlife, use of rodent/pest control, isolate sick animals and sharing habits of tools and equipment). The veterinarian’s primary practice type described in the survey followed the AVMA categories (i.e., food animal exclusive, food animal predominant, mixed animal, companion animal exclusive, companion animal predominant, equine and other) [26]. Given that most of these variables were not binary, they had to be modified for use by combining categories into two per variable. For satisfaction of veterinary care, *somewhat unsatisfied* and *not very satisfied* were categorized as *not satisfied*, and *somewhat satisfied* and *very satisfied* were categorized as *satisfied*. For the biosecurity questions, *never* and *rarely* were categorized as *no*, and *sometimes*, *usually* and *always* were categorized as *yes*, unless stated otherwise. Four exposure variables were considered: location (state), setting (urban, peri-urban or rural), usage of animal products (personal, commercial, or both) and animal type. Given that an owner could have several animal types on premises, this variable was made into 5 dummy ‘yes’ variables for cattle, small ruminants, poultry, swine and horses. Exploratory univariate analysis was conducted, and then multivariate models were constructed. Model selection was done first via automated stepwise selection based on Akaike Information Criteria (AIC) and then checked using manual forward selection process considering AIC and p-values from both the likelihood ratio tests (LRT) between models, and the Wald test within model. A p-value of 0.10 was set as the significance level to account for the small sample size for some questions.

## Results

### General demographics

**Fig 1:**
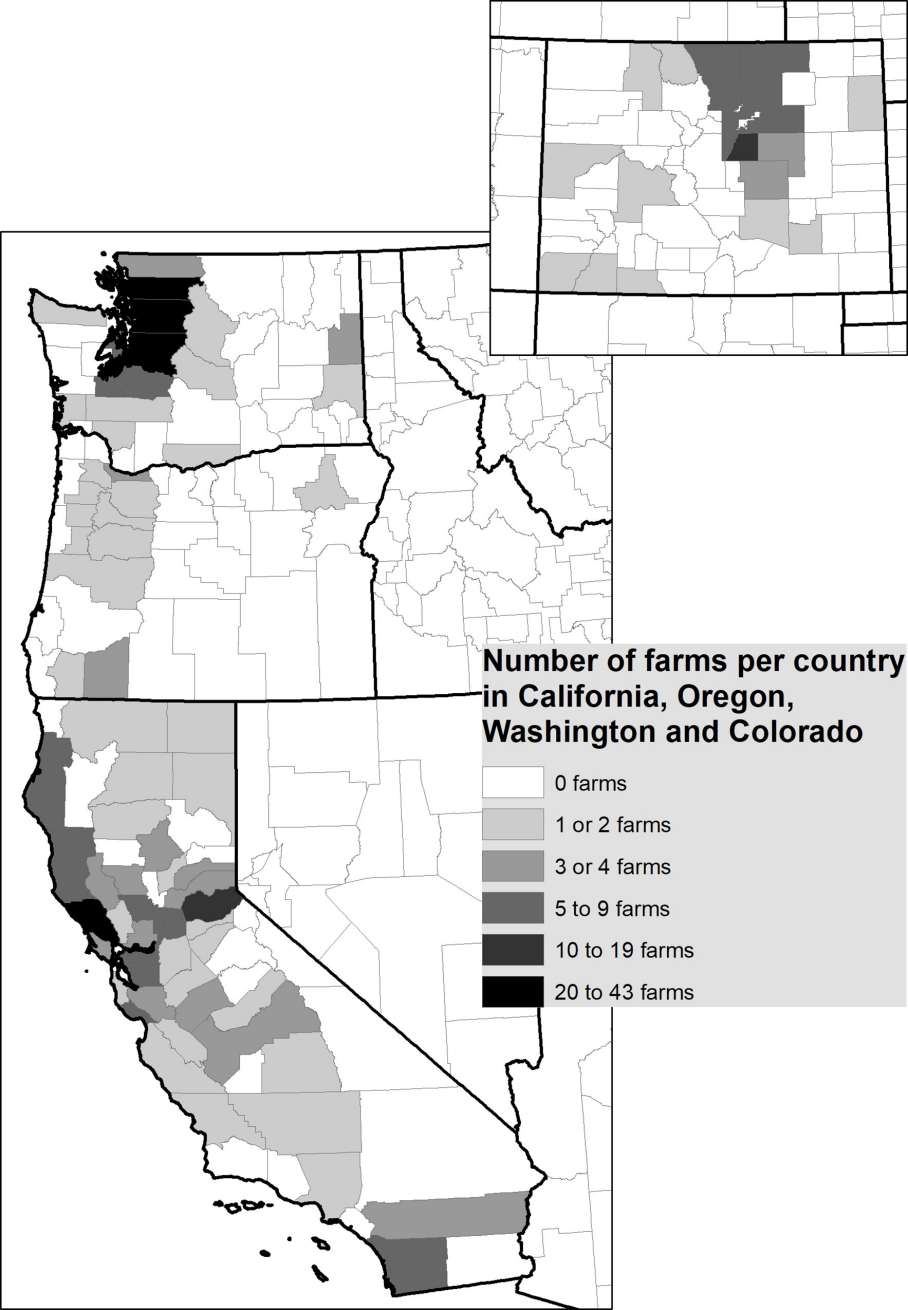
Distribution of animal owners (n = 417) surveyed by counties in 4 western states (Washington, Oregon, California and Colorado).

**Fig 2:**
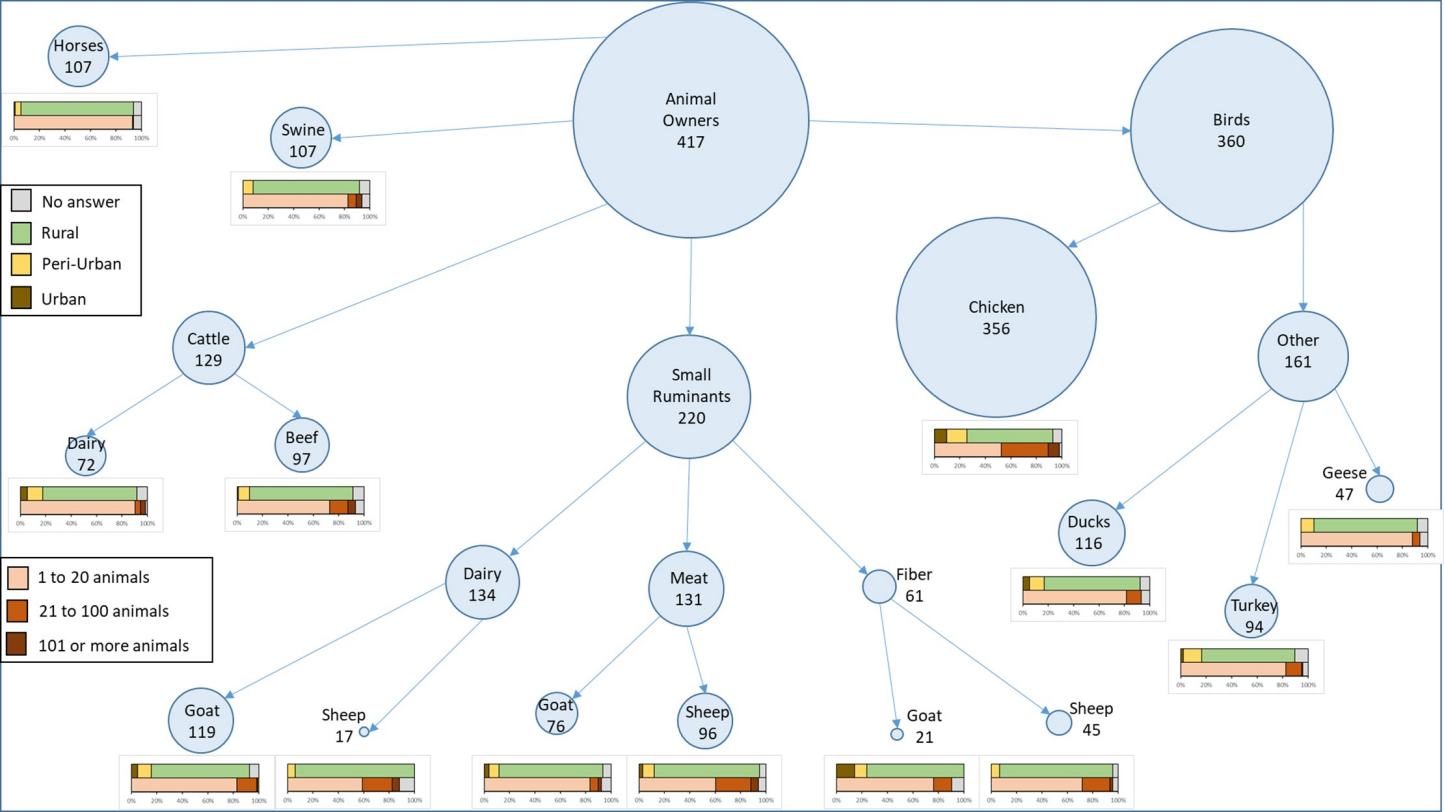
Number of small-scale livestock and poultry owners (n = 417) owning each type of animal (birds, cattle, small ruminants, swine and horses), herd size and settings (urban, peri-urban and rural) by type of animal.

A total of 435 surveys (394 online and 41 hardcopies) were completed or partially completed. Respondents had premises located in 16 states and 103 counties. These were located mainly in Washington state (156 premises in 19 counties), California (162 premises in 41 counties), Colorado (78 premises in 18 counties) and Oregon (21 premises in 12 counties), with 13 premises in 12 other states and 5 no answers. The surveys from states other than the 4 western states (CA, CO, OR and WA) and those providing no answers were excluded from all descriptive analysis (Fig 1). Most respondents considered their premise as rural (76.4%, 284/386). The most common animals owned were chickens (86.4%, 356/412) followed by small ruminants (53.0%, 220/412) and cattle (31.3%, 129/412) (Fig 2). Twenty-seven percent of owners (109/409) kept only one species, 26.9% (110/409) had two species, and 46.5% (190/409) had three or more species, with 62.8% (257/409) having a mix of mammals and birds (Table 1). Most respondents said their animal products were for personal consumption (73.8%, 301/408) (Table 1). For most species, herd or flock size was small, with more than 80% of respondents having fewer than 20 animals of a given species, except for chickens and sheep where 25% and 47% of respondents had more than 20 animals (Fig 2). The most common products sold by respondents were eggs (77.5%, 241/311) and meat (57.9%, 180/311) (Table 1). Most premises had less than $10,000 in sales in the year prior to the survey (76.4%, 240/314) (Table 1). Owners used a wide variety of marketing channels to sell their products, with the most common being the internet (34.7%, 96/277) and farmers’ markets (24.9%, 69/277) (Table 1).

**Table 1:** General demographics of survey respondents (n = 417) on surveyed premises in 4 western states (Washington, California, Colorado and Oregon)^a^.

Question	Number	% of total responses
**What state are you in?**		
Washington	156	37.4
California	162	38.8
Colorado	78	18.7
Oregon	21	5.0
**What type of area is your property in?**		
Rural	284	73.6
Peri-urban & Suburban	64	16.6
Urban	38	9.8
No answer	31	
**Do your neighbors raise livestock?**		
Yes	316	77.1
No	94	22.9
No answer	7	
**Number of species raised**^**b**^		
1	109	26.7
2	110	26.9
3	91	22.2
4	58	14.2
5	32	7.8
6	9	2.2
**What is the use of your livestock?**		
Personal consumption	301	73.8
Pets	137	33.6
Sale of animal products	195	47.8
Sale of live animals	137	33.6
Other	38	9.3
No answer	9	
**What type of product do you sell?**		
Eggs	241	77.5
Meat	180	57.9
Milk and dairy products	31	10.0
Value-added food products	35	11.3
No answer	106	
**What was your gross total sales last year?**		
Less than $10,000	240	76.4
$10,000 to $50,000	49	15.6
More than $50,000	25	8.0
No answer	103	
**Which marketing channels have you used for animal and animal product sales in the last 12 months?**		
Internet sales	96	34.7
Farmers’ markets	69	24.9
Farm stands	43	15.5
Community-supported agriculture	38	13.7
Wholesale	39	14.1
Retail	35	12.6
Restaurants	31	11.2
Other	161	58.1
None of these or no answer	140	

^a^ Due to variation in completion of the surveys, relative frequencies of responses were calculated on per-question basis, using total respondents for each question as the denominator. Many questions allowed for the selection of more than one response, thus the total number of responses may exceed total number of respondents (n) per question.

^b^ Species is used here in a broad sense, and groups of similar animals were grouped together: ducks and geese; chickens and turkey; goats and sheep. Other species included horses, swine and cattle.

### Animal health and husbandry knowledge and perceptions

**Fig 3:**
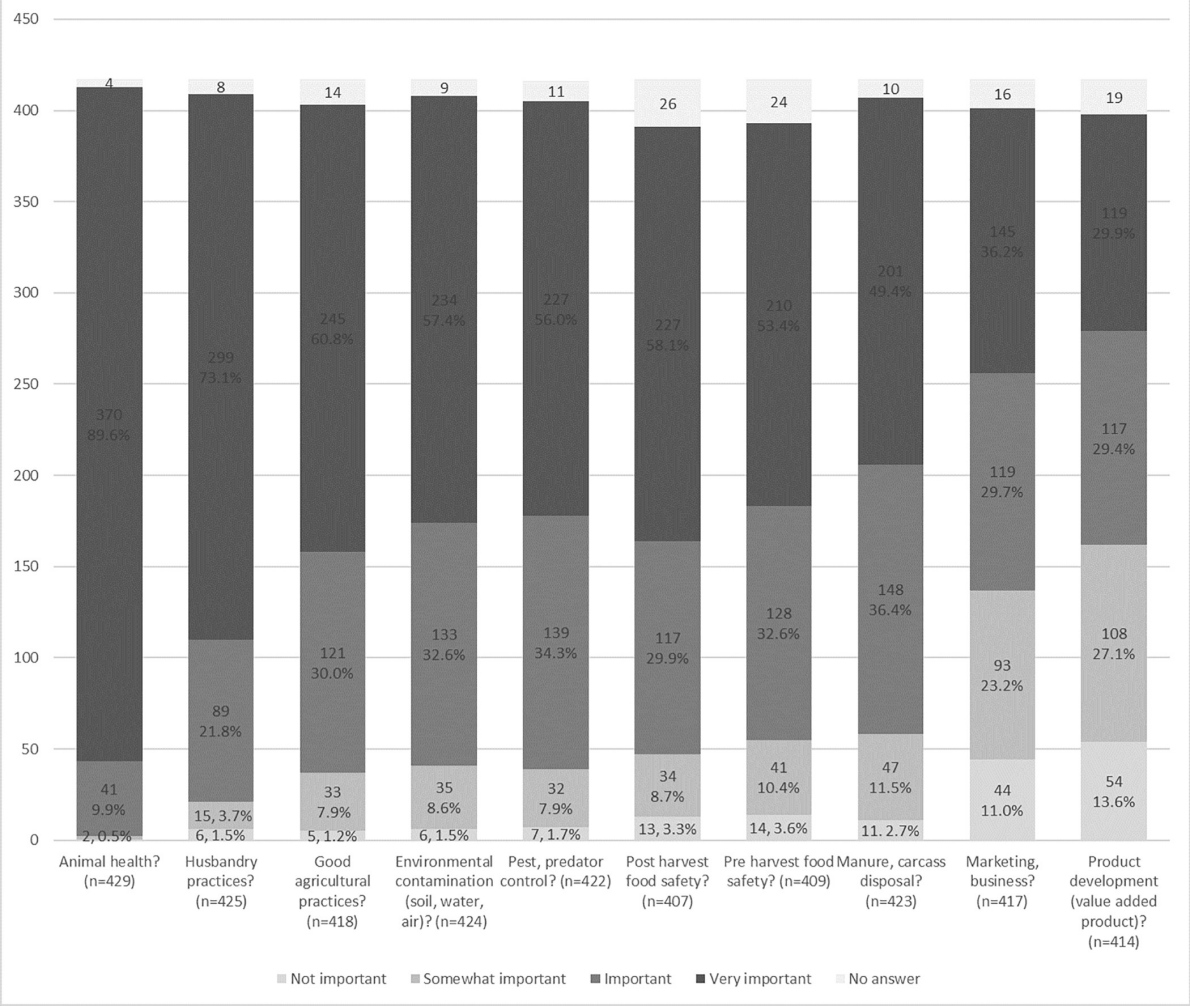
Perceptions of animal health, husbandry practices, pest control, food safety and marketing by small-scale livestock and poultry owners in 4 western states (Washington, California, Colorado and Oregon). *Footnote*: Proportions are given as the valid percentage: the percentage only of individuals who answered the question, and do not count no answers).

A series of questions looked at perceptions of owners about different aspects of animal health and husbandry, biosecurity (e.g., environmental contamination, rodent and pest control, manure and carcass disposal), food safety practices and marketing (Fig 3). In most cases, a large majority of respondents agreed that a given practice was important to very important (85.7% to 99.5%). Marketing and product development were less unanimously deemed important but were still considered so by a majority of respondents (65.8%, 264/401, and 59.3%, 236/398) (Fig 3).

**Fig 4:**
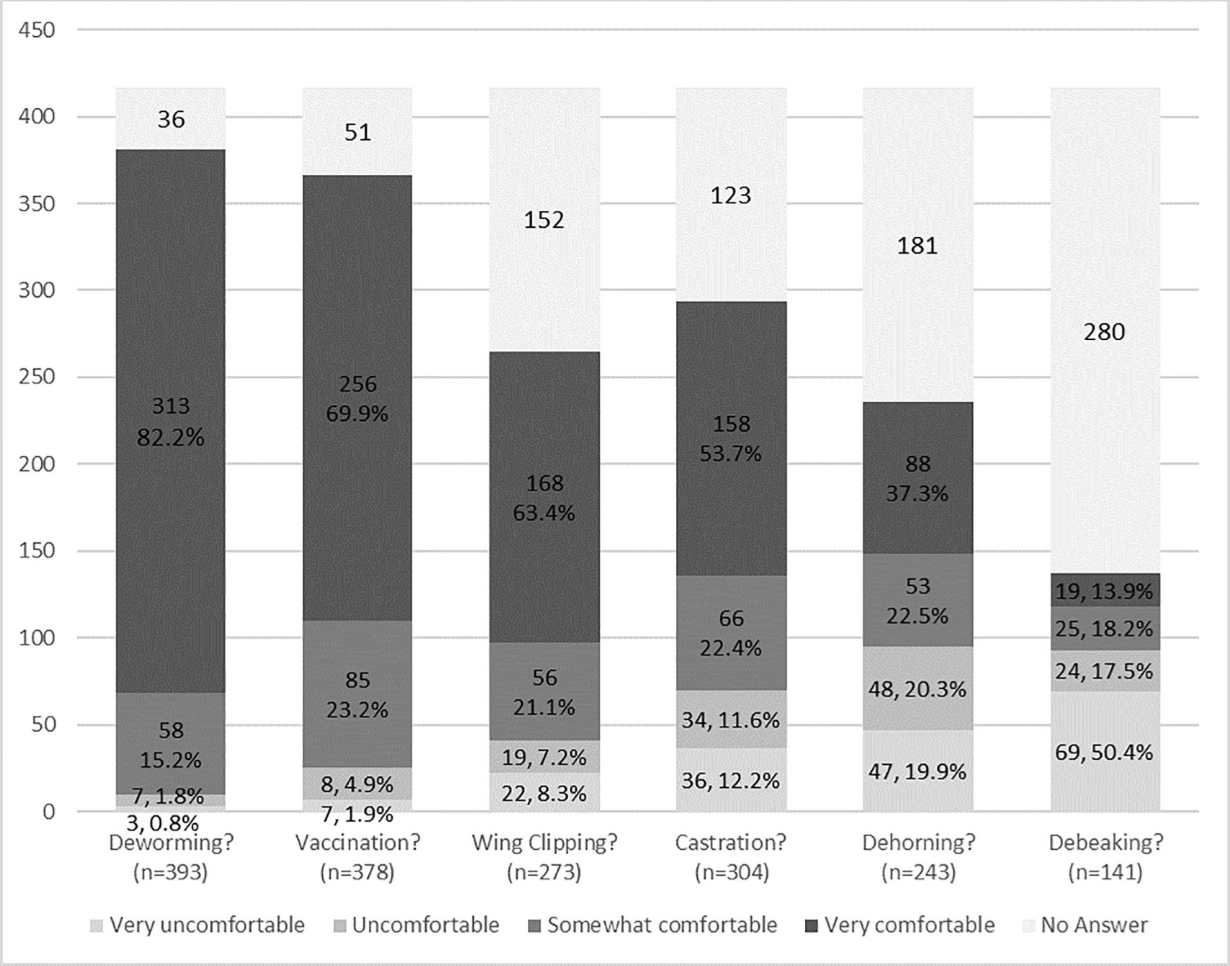
Self-assessment of competence at owner-performed health-related interventions reported by small-scale livestock and poultry owners in 4 western states (Washington, California, Colorado and Oregon). *Footnote*: Proportions are given as the valid percentage: the percentage only of individuals who answered the question, and do not count no answers).

Owners were asked to assess their level of competence for a number of animal health interventions performed on animal premises (Fig 4). Respondents were most comfortable with prevention-related interventions: 97.4% (371/381) were at least somewhat comfortable deworming and 93.2% (341/366) were at least somewhat comfortable vaccinating. With regard to surgical interventions, a majority of respondents were at least somewhat comfortable with wing clipping (84.5%, 224/265), castration (76.2%, 224/294) and dehorning (59.7%, 141/236). However, far fewer respondents felt comfortable with debeaking (32.1%, 44/137) (Fig 4).

When looking for animal health information, the main source of information was the internet (81.8%, 338/413), followed by veterinarians (61.5%, 254/413) and friends and neighbors (47.0%, 194/413). A plurality of respondents sought information on a monthly basis (48.1%, 193/401) (Table 2). For information on how to perform treatments and procedures, the main sources of information were the same: internet (70.7%, 290/410), veterinarians (58.0%, 238/410), and friends and neighbors (41.7%, 171/410). However, this type of information was sought less often, with a plurality doing so on a yearly basis (45.2%, 177/392) (Table 2).

**Table 2:** Knowledge and information sources of small-scale livestock and poultry owners in 4 western states (Washington, California, Colorado and Oregon).

Question	Number (%)	Number (%)
	Animal health	Animal treatment and procedures
**Where do you get information from?**		
Internet	338 (81.8)	290 (70.7)
Veterinarian	254 (61.5)	238 (58.0)
Friends/neighbors	194 (47.0)	171 (41.7)
Extension agent	140 (33.9)	70 (17.1)
Feed store	112 (27.1)	77 (18.8)
Other	80 (19.4)	81 (19.8)
No answer	4	7
**How often do you seek this information?**		
Daily	29 (7.2)	11 (2.8)
Weekly	98 (24.4)	52 (13.3)
Monthly	193 (48.1)	163 (41.6)
Yearly	115 (28.7)	177 (45.2)
Never	6 (1.5)	12 (3.1%)
No answer	16	25

Of 277 respondents who answered a question about training for themselves or their staff, a large majority mentioned receiving training in animal health (70.5%, 223/277) and sanitation and cleaning (70.8%, 196/277). A narrow majority received training in biosecurity (58.1%, 161/277) and a minority received training in post-harvest (45.7%, 125/277) and pre-harvest food safety (37.9%, 105/277).

### Access to veterinary care

**Fig 5:**
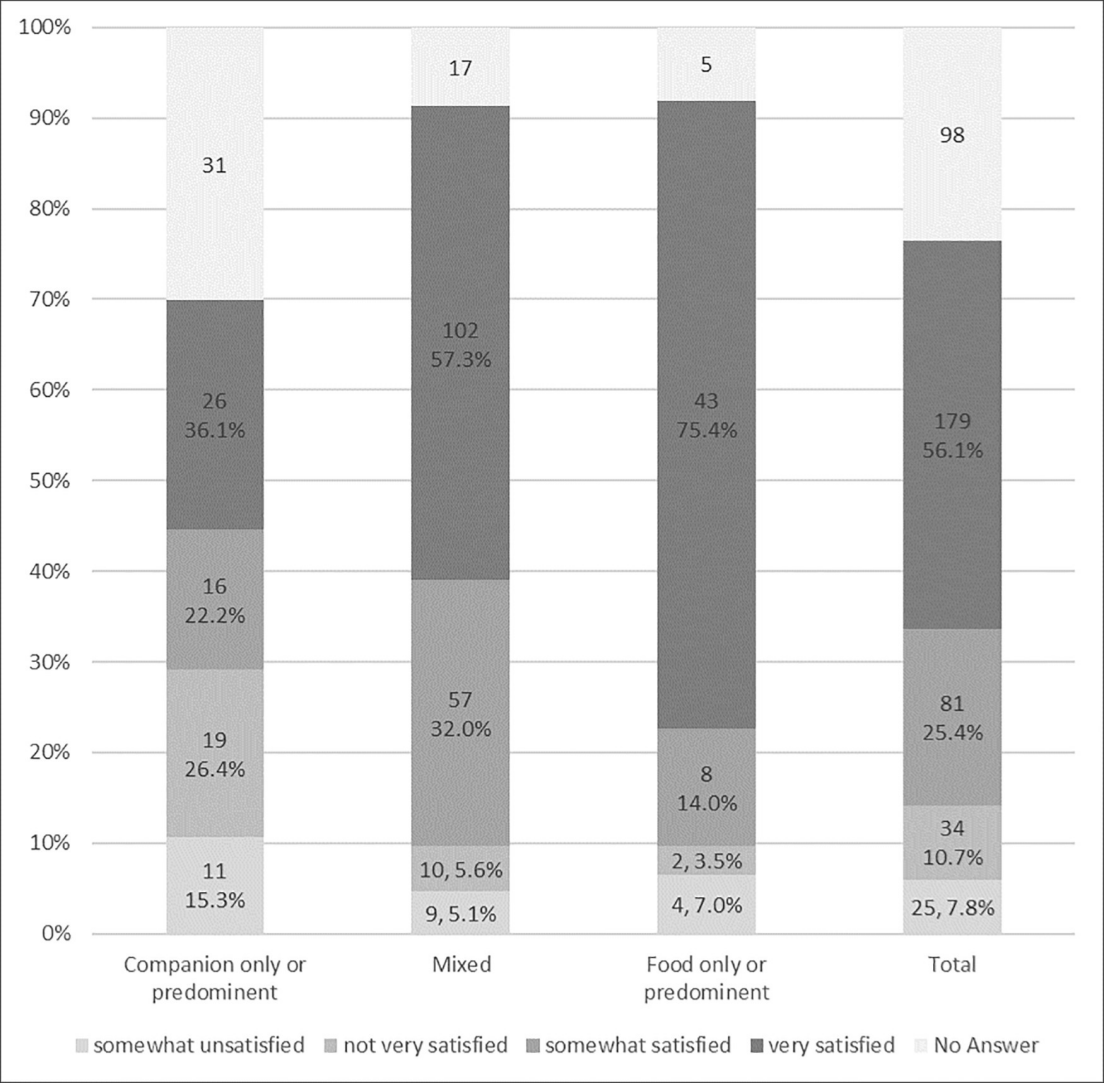
Satisfaction level of small-scale livestock and poultry owners by type of veterinarian practice in 4 western states (Washington, California, Colorado and Oregon). *Footnote*: Proportions are given as the valid percentage: the percentage only of individuals who answered the question, and do not count no answers).

Most respondents (75.8%, 316/416) said they knew of a veterinarian in their area that treated livestock (Table 3). When asked about the type of practice of their veterinarians, 50.3% (195/388) had access to mixed practices, 26.5% (103/388) had access to companion animal–predominant practices, and 16.0% (62/388) had access to food animal–predominant practices (Table 3). Overall, the majority of respondents felt satisfied with the level of veterinary care they had received (81.5%, 260/319) (Table 3). However, more owners using a food animal–predominant or mixed practice veterinarian were satisfied (89.5%, 51/57, and 89.3%, 159/178) compared to owners with a companion animal veterinarian (58.3%, 42/72) (Fig 5). Although most of the respondents stated that they knew of a local veterinarian who was able to treat their poultry/livestock, most of the owners had not sought veterinary care in the previous 12 months. Of the 351 owners who reported that they had an animal health concern in the past 12 months, only 148 (42.7%) called a veterinarian for help. Another 46 individuals reported not having had any animal health problem in the previous 12 months.

**Table 3:** Access to veterinary care of small-scale livestock and poultry owners in 4 western states (Washington, California, Colorado and Oregon).

Question	Number	% of total responses
**Do you know of a veterinarian in your area that treats livestock?**		
Yes	316	75.8
No	101	24.2
No answer	1	
**What is your veterinarian's primary practice type?**		
Companion animal only or predominant	103	26.5
Mixed animal (food and companion animals)	195	50.3
Food animal only or predominant	62	16.0
None of these	28	7.2
No answer	29	
**How satisfied are you with the level of veterinary care of your livestock?**		
Somewhat unsatisfied	25	7.8
Not very satisfied	34	10.7
Somewhat satisfied	81	25.4
Very satisfied	179	56.1
NA or no answer	98	

The most common health problems for owners who sought veterinary help were infectious and parasitic diseases (25.7%, 38/148), internal medicine issues (21.6%, 32/148), reproductive and neo-natal health issues (17.6%, 26/148), trauma (9.5%, 14/148), locomotion issues (6.1%, 9/148), and diseases/deaths of unknown causes (6.8%, 10/148). For individuals who did not seek veterinary help and therefore did not have a professional diagnosis made, these owners believed that the health issues involved infectious and parasitic diseases (29.0%, 60/207), diseases/deaths of unknown causes (15.0%, 31/207), internal medicine issues (14.0%, 29/207), reproduction and neo-natal health (12.1%, 25/207), trauma (12.1%, 25/207), and locomotion issues (6.8%, 14/207). It was noticeable that for most issues, owners were evenly divided between calling for a veterinarian or not. However, there are a few cases in which that wasn’t the case. When faced with deaths of unknown causes and traumatic injuries, owners were less likely to call a veterinarian: 25% (10/41) of deaths/disease from unknown causes and 36% (14/39) of traumas led to a veterinary call. The overall category of infectious/parasitic diseases was the most common problem encountered by both owners seeking veterinary help or not. It is important to note that of 79 owners faced with infectious diseases and internal parasites, 36 (45.6%) sought veterinary help, whereas of 19 owners faced with ectoparasites only 2 (10.5%) sought veterinary care.

The most commonly reported reason for not calling the veterinarian following the previously mentioned health problems was being able to manage the problem on their own (45.3%, 86/190). This was especially true in cases with recurring issues like ectoparasites, when individuals applied the treatment they had used in the past. Cost was the second most reported reason (21.6%, 41/190), with 22 specifying cost in relation to the problem being in poultry, a low-cost animal. Next came the animal dying (11.1%, 21/190) or the owner culling it immediately (8.4%, 16/190). Finally, other reasons mentioned were lack of veterinary experience for the species in question, lack of availability of a veterinarian, lack of time, no evidence of spread of the problem, or the owner didn’t consider it necessary.

In a similar question asking about what might drive the decision to call a veterinarian or not, in general, the most important consideration mentioned was cost (48.3%, 187/387), followed by availability of veterinarians with livestock experience (16.3%, 63/387), the experience and knowledge of the owner (10.1%, 39/387) and the distance from available help (9.5%, 37/387). Other minor reasons were illness severity and outcome, potential spread, animal welfare, the need for advice, and personal contact and trust with the veterinarian.

Results from the multivariate logistic regression showed that knowing a veterinarian in their area who treated livestock was significantly associated with the setting (peri-urban and rural) of the premise and the ownership of cattle, small ruminants, and birds (Table 4). Peri-urban owners were less likely to know a veterinarian who treats livestock (odds ratios (OR) = 0.51), while for urban owners there was a trend for being less likely to know a veterinarian who treats livestock (OR = 0.50), when adjusted for other variables (Table 4). Poultry owners had 0.28 times the odds (OR = 0.28) of knowing a veterinarian who treats livestock (Table 4). On the other hand, owners of cattle and small ruminants had higher odds of knowing such a veterinarian (OR = 2.32 and 2.89, respectively) (Table 4).

**Table 4:** Final multivariate logistic regression models investigating the association between veterinary care and biosecurity practices of urban, peri-urban livestock owners and backyard owners in 4 western states (Washington, California, Colorado and Oregon)^a^.

Outcome	Exposure variable	Level	Yes (%)	Odds-Ratio	95% Confidence interval	p-value (Wald test)
**Knows of a veterinarian in the area that treats livestock (Yes/No) (n = 373)**	Setting	Rural	81.2	Reference		
		Peri-urban	61.0	0.51	0.27 to 0.96	0.038
		Urban	56.8	0.50	0.23 to 1.09	0.080
	Cattle	No	70.8	Reference		
		Yes	86.7	2.32	1.22 to 4.38	0.010
	Small ruminants	No	63.9	Reference		
		Yes	85.3	2.89	1.71 to 4.89	<0.001
	Birds	No	93.0	Reference		
		Yes	73.3	0.28	0.08 to 0.96	0.043
**Primary practice type of owner’s veterinarian (Mixed or food animal /Companion) (n = 322)**	Setting	Rural	75.7	reference		
		Peri-urban	54.8	0.49	0.24 to 1.02	0.056
		Urban	48.3	0.51	0.22 to 1.21	0.13
	Cattle	No	65.1	Reference		
		Yes	81.7	2.09	1.13 to 3.84	0.018
	Small ruminants	No	57.8	Reference		
		Yes	79.7	2.62	1.54 to 4.45	<0.001
	Birds	No	97.6	Reference		
		Yes	66.5	0.06	0.01 to 0.43	0.006
**Satisfaction level of animal owners of the veterinary care received (Satisfied/ Not Satisfied) (n = 284)**	Location	California	80.0	Reference		
		Colorado	67.2	0.44	0.20 to 0.94	0.035
		Oregon	68.8	0.44	0.13 to 1.51	0.19
		Washington	89.1	2.01	0.92 to 4.43	0.082
	Use	Personal	82.8	Reference		
		Both	81.6	0.89	0.43 to 1.84	0.75
		Commercial	72.0	0.43	0.18 to 1.05	0.064
	Cattle	No	77.0	Reference		
		Yes	86.6	2.16	1.03 to 4.51	0.041
	Horses	No	77.8	Reference		
		Yes	86.0	2.06	1.98 to 4.32	0.057
**Allows contact between wildlife and livestock (Yes/No) (n = 363)**	Small ruminants	No	57.0	Reference		
		Yes	43.9	0.59	0.39 to 0.90	0.014
**Isolates sick animals from healthy ones (Yes/No) (n = 365)**	Use	Personal	80.5	Reference		
		Both	87.4	1.91	1.01 to 3.63	0.046
		Commercial	74.2	0.73	0.35 to 1.50	0.39
	Small ruminants	No	87.4	Reference		
		Yes	78.8	0.48	0.27 to 0.86	0.014
**Uses regular pest control on animal premise (Yes/No) (n = 365)**	Use	California	49.6	Reference		
		Colorado	65.3	2.02	1.11 to 3.67	0.021
		Oregon	47.6	0.89	0.35 to 2.26	0.81
		Washington	59.1	1.50	0.93 to 2.44	0.099
	Swine	No	52.6	Reference		
		Yes	66.3	1.87	1.14 to 3.07	0.013
**Shares tools and equipment with neighbors (Yes/No) (n = 369)**	Cattle	No	19.5	Reference		
		Yes	36.6	2.39	1.46 to 3.91	<0.001

^a^In these questions, “sometimes” was categorized as “Yes” when making the outcome binary.

The type of veterinary practice used by respondents was associated with the setting and ownership of birds, cattle and small ruminants. Bird owners had 0.06 times the odds (OR = 0.06) of having a mixed or production-oriented veterinarian compared to a companion one (Table 4). On the other hand, cattle and small ruminant owners were more likely to have a production/mixed veterinarian (OR = 2.09 and 2.62, respectively) (Table 4). For owners in peri-urban settings, there was trend for a lower odds of having such a veterinarian compared to rural owners (OR = 0.49) (Table 4).

The level of satisfaction with veterinary care received was associated with location, use of animals, and ownership of cattle. Owners in Colorado were significantly less likely to be satisfied with the veterinary care they received (OR = 0.44) compared to owners in California (Table 4). For owners using their animals for commercial use (OR = 0.43), there was trend to be less likely to be satisfied by the veterinary care received than those having animals for personal use (Table 4). Finally, cattle owners had higher odds of being satisfied (OR = 2.16) compared to those who did not own cattle (Table 4).

### Biosecurity practices and pre- and post-harvest food safety practices

**Fig 6:**
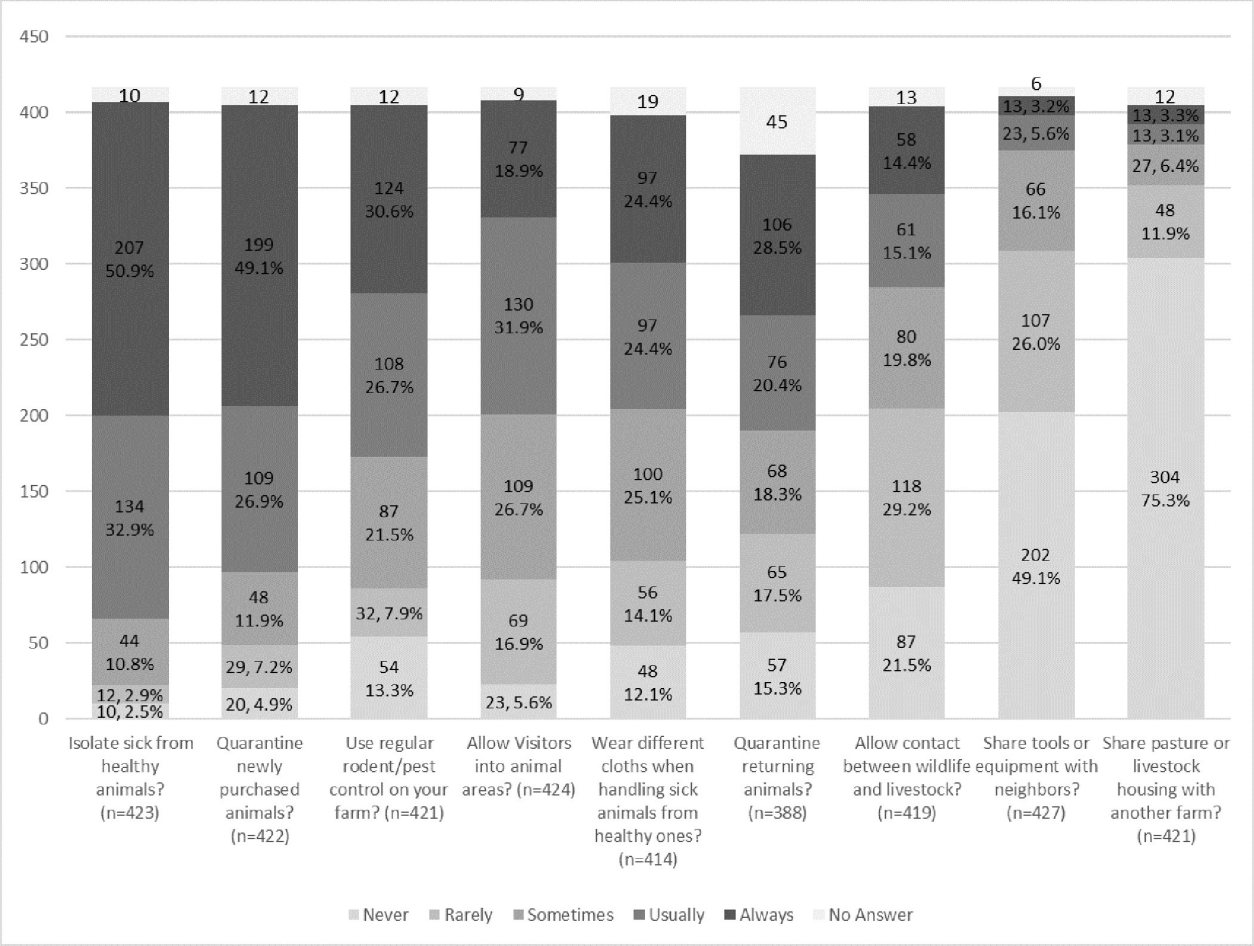
Biosecurity practices among small-scale livestock and poultry owners in 4 western states (Washington, California, Colorado and Oregon). *Footnote*: Proportions are given as the valid percentage: the percentage only of individuals who answered the question, and do not count no answers).

When looking at on-premise biosecurity practices (Fig 6), 83.8% (341/407) of respondents said they usually or always isolated sick animals from healthy ones, 76.0% (308/405) said they usually or always quarantine newly purchased animals, and 48.9% (182/372) said the same for returning animals. Use of rodent/pest control was common in 57.3% (232/405) of respondents and wearing different clothing when handling sick versus healthy animals was common in 49.5% (194/398) of respondents. Most respondents did not participate in sharing tools and pastures with other owners: 75.2% (309/411) never or rarely shared tools and 86.9% (352/405) never or rarely shared pastures. However, fewer respondents limited visitors in animal areas and contact between wildlife and animals, with 50.7% (205/404) never or rarely allowing contact with wildlife and 22.5% (92/408) never or rarely allowing visitors.

For post-harvest food safety and hygiene practices, the large majority of respondents said they usually or always handle food products in areas separate from animal facilities (87.9%, 313/356) and use disinfectants (81.3%, 300/369). Fewer, however, had clothing rules, with 66.1% (240/363) saying they wear different clothing when handling food or animals, 50.3% (182/362) changing clothes when handling food products from animals, and 35.8% (132/369) wearing gloves when preparing food products from animals.

Logistic regression results showed that quarantine of new animals was not significantly associated with any of the variables considered in the models. However, isolating sick animals was significantly associated (OR = 0.48, 95% CI = 0.26 to 0.81) with small ruminant ownership when adjusted for type of use of animal products (Table 4). Owners who used their animals for both personal and commercial use were more likely to isolate sick animals compared to personal-only users (OR = 1.91), but there was no significant evidence of commercial animal owners differing from personal-only users (Table 4).

Allowing contact of livestock with wildlife was associated with ownership, small ruminants owners were less likely to allow contact (OR = 0.59) (Table 4). Use of rodent/pest control was significantly associated with swine ownership, with swine owners being more likely to use pest control (OR = 1.87) (Table 4). Owners in Colorado were more likely to use rodent/pest control than owners in California (OR = 2.02). Owners in Oregon and Washington did not have different rodent/pest control use compared to owners in California. Finally, tool and equipment sharing were associated with ownership of cattle. Cattle owners were more likely to share tools (OR = 2.39) (Table 4).

## Discussion

This cross-sectional study described small-scale and backyard livestock and poultry owners in four western states (California, Oregon, Washington State and Colorado). The present study characterized animal health and husbandry, identified potential veterinary service needs of these animal owners in the western U.S., and assessed management practices with regards to disease prevention and attitudes about animal health. This study differed from other studies that focused on the regulatory aspect of raising animals in urban and peri-urban areas [1,20]. In our study, chicken and small ruminants were the most common animals reported, with a relatively small herd size (less than 20 animals) and a wide range of species.

More than 70% of the respondents reported that they owned multiple species of animal (71%) and described themselves as living in rural areas (73.6%). In a recent study conducted in eastern Australia, about one third of backyard and small-scale pig producers lived in peri-urban areas and the remainder in rural areas; more than 80% kept ruminants and domestic birds [27]. Similarly, Gillespie et al (2015) reported that small-scale pig holders in England owned poultry (74.5%) and sheep (48.4%) in addition to dogs and cats (79.3%) [18]. A concern with mixed farms (i.e., with different animal species) is that they may be more prone to disease transmission and animal contacts, and therefore pose a unique risk to introduction and spread of endemic and exotic diseases [18,27,28].

In recent years, several studies have focused on urban and backyard poultry flocks in North America [9,14,15,19,21,29] but few have characterized other non-poultry backyard and small-scale livestock owners [20]. In the present study, most respondents utilized their animals’ products for their own consumption but about half sold animal products (eggs and meat), primarily through internet sales or farmers’ markets with sales less than $10,000 a year. Similarly, Elkhoraibi et al (2014) reported that the main reason for keeping chickens was food for home use (95% of backyard chickens in the U.S.) [14]. The growing interest in raising food animals for personal use has been reported by others in several U.S. regions, and many owners may consider the animals more as pets than as production animals [2,17,23]. In the present study, about one-third considered their animals as pets, similar to urban livestock owners surveyed in 48 cities [20]. The small-scale and backyard owners keep animals to ensure a better source of food, to be assured where their food comes from and how is it produced [14,20,28]. Moreover, they perceived their eggs and meat were higher quality, more nutritious, safer to consume and tasted better than those raised in commercial settings, and also perceived that their chickens were healthier and experienced better welfare [14,20,22]. However, our study identified two food safety concerns. First, not all owners had different clothing for handling animals and for handling animal products. Second, only a third of owners wore gloves when preparing food products from animals. If the latter owners were also selling those animal products, there could be some risk to consumers.

Small-scale and backyard livestock husbandry in western states is done with a wide range of species and faces significant barriers for veterinary care. Twenty-six percent reported their veterinarian’s practice type as companion animal but two-thirds were working with a mixed animal or food animal practice. Most owners in the survey were satisfied with the level of care. In a survey conducted in England, the majority of smallholder and pet pig owners consulted either mixed or farm-veterinary practices and were satisfied with the overall service that their veterinarian offered [18]. In contrast, the majority of small-scale pig holders in a study in Scotland reported that a veterinarian visited the pigs less than once per year or never [28]. Also, most of the owners of small poultry flocks in Canada did not use veterinary services for their flocks [19]. In the present study, the veterinarian’s primary practice type and the owner’s satisfaction with veterinary care were associated with state, species owned, use of animals, and setting. Bird owners (as opposed to cattle and small ruminant owners) located in peri-urban areas (compared to rural areas) were less likely to know of a veterinarian who treated livestock. Moreover, cattle owners were two times more likely to report being satisfied with the level of veterinary care for their animals. Small-scale and backyard owners in Colorado were less likely to be satisfied with the veterinary care that they received compared to owners in California. Because many owners claim a high level of comfort for performing common animal husbandry procedures such as preventive medicine (e.g., vaccination and deworming) and basic surgical (e.g., wing clipping, castration, and dehorning) interventions, they are likely to call a veterinarian only rarely and only in case of major health problems or surgery. Poultry owners reported that they were not comfortable performing debeaking as compared to other basic surgical interventions. This may had been overestimated, as “debeaking” and “beak trimming” were used interchangeably, and poultry sources were not accessed in this survey (beak trimming is commonly performed in the hatchery). The main reasons for not seeking veterinary care were being able to manage the problem on their own and cost. The decision to call a veterinarian was primarily influenced by cost, followed by availability of veterinarians with livestock experience.

Peri-urban owners (as compared to rural owners) and poultry owners (as opposed to cattle and small ruminant owners) were less likely to know a veterinarian who treated livestock. The access to limited veterinary care for these owners could be associated with lack of livestock/poultry-focused veterinary practices in certain states and geographic areas [30] and/or the specialization in small-animal/companion animals observed in a geographic area [26]. Veterinary clinics across the U.S. are primarily either companion animal–only or companion animal–predominant (62% of all clinics in the U.S.) [31]. Practices that provide substantial livestock animal services within the surveyed group of small-scale livestock producers could be over-represented [31], as most participants reported knowing a veterinarian who treats livestock in their area.

The majority of respondents who sought animal husbandry information utilized the internet. However, many poultry and livestock owners in UPA lack access to specific technical information and veterinary oversight. In the absence of veterinary oversight, there are potential animal health and welfare issues due to improper management and husbandry. Lack of proper diagnosis may result in inappropriate treatments leading to poor animal health and subsequent welfare issues as well as risks to human and public health due to zoonotic diseases or drug residues in the food chain [19]. This is particularly important regarding the potential misuse of antimicrobial and prohibited substances in livestock species, which may lead to noncompliance of withdrawal intervals, drug residues and potential development of antimicrobial resistance pathogens [19]. Moreover, recent changes in federal legislation (e.g., Veterinary Feed Directive) in the U.S. and in California legislation (SB-27) increased the importance of a valid VCPR for access to antimicrobials by small-scale and backyard livestock owners [12,13].

The diagnosis and control of infectious diseases is particularly important among small-scale and backyard livestock premises. A recent study of 41 backyard poultry flocks in California demonstrated a high prevalence of antibodies (between 45% and 97%) to avian respiratory pathogens (e.g., infectious bronchitis, *Mycoplasma synoviae* and *M*. *gallisepticum*), indicating their potential as a reservoir or amplifier for these diseases [29] and spill-over to commercial production systems. Disease outbreaks in commercial operations, such as virulent Newcastle Disease (vND, known as Exotic Newcastle Disease) and Marek’s Disease, have originated in backyard flocks and spread to commercial flocks [3,11]. The 2002–2003 outbreak of vND in California, originally confirmed in a California backyard flock, spread to commercial poultry operations [32]. The ongoing 2018 vND outbreak in backyard chicken premises [33] shows the potential for backyard poultry flocks to contribute to the transmission of diseases and put commercial poultry flocks at risk [29,32]. Ultimately, backyard and small-scale owners have responsibility for animal welfare and safety of the food produced, so they should seek veterinary services that will support rapid diagnosis of infectious diseases and provide medical care for these animals, as well as advice on husbandry and disease-prevention practices. In developed nations, it commonly falls to the local animal experts to assist and advise peri-domestic animal producers—largely companion-animal veterinarians in urban areas. Veterinarians can have a major role on detecting endemic and emerging diseases, and implement mitigation strategies to reduce disease transmission [28].

The present study identified variable percentages of self-reported compliance on biosecurity practices, which could potentially lead to disease transmission within animal premises and between premises. The majority of small-scale and backyard livestock and poultry owners reported that they isolated sick animals from healthy ones (83.8%) and kept newly purchased animals in quarantine (76.6%), but other biosecurity practices were reported at a lower rate, such as the quarantine of returning (e.g., from fairs, shows) animals (49%), rodent/pest control (57.3%), wearing dedicated clothes when handling sick animals (49.5%), avoiding livestock contact with wildlife (50.7%) or limiting visitors (22.5%). Moreover, livestock species type (cattle, small ruminants and swine), use (commercial), and state (Colorado) were associated with implementation of different biosecurity practices (wildlife contact, isolation of sick animals, pest control, and tools/equipment sharing). Similarly, other studies reported variable biosecurity practices compliance rates and associated risks in the U.S. [14,15,21,29], United Kingdom [17,18,28], Canada [19], and Australia [27]. Derkesen et al (2017) reported that backyard poultry owners lack biosecurity measures such as dedicated shoes, and they use unreliable chicken sources [29]. Moreover, Elkhoraibi et al (2014) reported biosecurity measures were influenced by the motivation for keeping chickens (as food or pets) [14]. In present study, small ruminant owners were less likely to isolate sick animals compared with non-small ruminant owners. Transmission of diseases between wildlife and domestic animals may pose a burden in terms of animal and public health [34–36].

In the present study, bird ownership was not associated with reported contact with wildlife, isolation of sick animals, use of regular pest control, or equipment sharing. The potential risk of backyard poultry contact with wild birds is the transmission of zoonoses such as *Salmonella* and HPAI. Specifically, avian influenza information is highly relevant to small-scale and backyard poultry owners. As shown during the 2014–15 HPAI-H5N1 outbreak, many states have significant vulnerabilities and risks to both domesticated and wild bird populations. Due to the transient nature of wildlife and the geographic trends of migratory waterfowl (Washington State, Oregon, California and parts of Colorado lie directly in the Pacific flyway), there is no mitigation strategy available to eliminate all risks for HPAI outbreaks. This emphasizes the importance of creating and providing reliable sources of information and prevention strategies to those backyard flocks at high risk of contact with wildlife and proximity to commercial flocks [14,29]. Several education and outreach efforts have been undertaken by state and federal agencies and extension departments since the 2014–15 HPAI outbreak [37]. Moreover, with regard to the introduction and spread of an exotic and endemic diseases, small-scale and backyard owners are considered by veterinary practitioners, governmental officials and regulators to be a high-risk sector and pose a threat to commercial food animal operations [18,27,28]. Information on characteristics of small scale and backyard production and biosecurity protocols is therefore of value to federal and state agencies to adapt their emergency plans in case of exotic diseases, as well as to local agencies and veterinarians to adapt their control and surveillance programs for endemic diseases [28] and for extension specialists and educators to develop training and educational materials.

The growing number of urban animal and backyard premises in urban and peri-urban areas across the nation have triggered new ordinances, and many cities are modifying regulations to legally accommodate livestock and poultry in urban and suburban settings [20,24]. Local urban ordinances commonly regulate housing design and size, setbacks, maximum number of animals, sex, and species that can be kept in certain neighborhoods [20,24,38]. Recent studies have identified the need to extend those ordinances to animal health and welfare in order to protect the livestock industry and public health [20,24,38]. In a recent study of backyard poultry in Colorado, few municipalities required any regulations related to animal health, feed, welfare, vaccinations or veterinary care [24]. As UPA livestock become more popular and new ordinances are created and/or reviewed to allow poultry and livestock in urban, peri-urban, and suburban settings, they should include specifics regarding the herd/flock size, manure management, slaughter and disposal, veterinary care, and owner/consumer education; also, households participating should be registered [20,38]. This may increase practice opportunities for companion animal veterinarians. If they broaden their knowledge/expertise to livestock species, they could play a key role in detection, prevention, diagnosis, and treatment of re-emerging and zoonotic diseases. As the number and variety of peridomestic livestock owners increases, veterinary medical professionals must be prepared and able to meet these animals’ medical needs and any public health issues that may arise.

Limitations of this study include the self-reported nature of the data and the fact that although the survey target was peri-urban to urban areas, more than 70% of the respondents reported living in rural areas. No specific definition of peri-urban and urban was provided in the survey, leaving the participants to make their own assessment. The respondents may have mis-classified the location of their premises, because categorization between urban, peri-urban, or suburban may be subjective and depend on many parameters [20]. Another limitation of this study is the recruitment strategy and the impossibility of assessing the response rate because the total number of small-scale and backyard livestock owners in the 4 states is unknown. Second, there is the possibility of self-report bias, as respondents might underreport certain behaviors or practices considered not the best practices by veterinarians and/or researchers. Third, this was an English-only survey circulated via email, listservs, social media, and at certain events (e.g., fairs, conferences, workshops): the data collected in this survey may overrepresent English speakers and people with internet access. In a cross-sectional study in four large cities (Denver, CO; Los Angeles, CA; Miami, FL; and New York City, NY) conducted by the United States Department of Agriculture’s (USDA) National Animal Health Monitoring System (NAHMS), Spanish-speaking participants were less likely to be aware with the risks of direct contact and *Salmonella* infection [21]. Therefore, because assessment of all non-English-speaking small-scale and backyard owners in the four western states is not possible, the extrapolation to Spanish-speakers should be done with care. The self-report bias and small sample in this study reduces external validity for inferring what non-responders, and the small-scale and backyard livestock owner population at large, think about their access to veterinary care in urban areas.

## Conclusions

Small-scale, urban/peri-urban livestock agriculture and peridomestic poultry ownership in particular are a growing trend. Determining needs and barriers for small-scale and backyard livestock owners has not been widely explored and, specifically, how veterinary professionals can better serve this community is poorly understood. Previous studies have shown that practitioners both desire and benefit from species-specific Continuing Veterinary Medical Education (CVME) training [39–41]. As a potential modality to improve veterinary care and public health for small-scale and backyard livestock owners and backyard poultry in particular, CVME programs may be an under-utilized mitigation strategy. Specific opportunities for the veterinary profession are to identify local or regional veterinary service needs for these owners, become equipped to address exotic or zoonotic disease detection and husbandry questions, and provide medical care as well as food safety advice.

## Supporting information

S1 File Questionnaire data on small-scale and backyard livestock owners in the western US. (XLSX)
